# Examining an integrated path model of psychological and sociocultural predictors of camouflaging in autistic adults

**DOI:** 10.1177/13623613241262110

**Published:** 2024-07-27

**Authors:** Sici Zhuang, Mackenzie Bougoure, Dawn-Joy Leong, Lydia Dean, Susan Reddrop, Kristin Naragon-Gainey, Murray Maybery, Diana Weiting Tan, Iliana Magiati

**Affiliations:** 1 School of Psychological Science, The University of Western Australia, Australia; 2 Independent Researcher, Singapore; 3 Autistic Advisor, Australia; 4 Independent Researcher, Australia; 5 Macquarie University, Australia

**Keywords:** autism, camouflaging, masking, psychosocial factors, social model of disability

## Abstract

Camouflaging involves various strategies employed by autistic individuals to modulate the visibility of their autistic characteristics in social situations and is thought to be primarily motivated by psychosocial factors. Most studies to date have examined a limited number of psychosocial factors related to camouflaging. This study tested an integrated model encompassing several hypothesised individual psychological (i.e. fear of negative evaluation, self-esteem, autistic identity) and broader sociocultural predictors (i.e. perceived stigma, vulnerability events, cultural tightness-looseness, independent and interdependent self-construal). Participants were 225 autistic adults (18–77 years; 60.9% female), who completed self-report measures online. Path analysis partially supported the model, explaining about 25% of the variance in camouflaging. Several sociocultural factors – perceived stigma, cultural tightness and independent and interdependent self-construal – were indirectly associated with camouflaging through individual psychological factors. Notably, greater vulnerability to negative life events was the only sociocultural factor directly contributing to more camouflaging, without mediation by individual psychological factors. Fear of negative evaluation emerged as a strong predictor, mediating most relationships between broader sociocultural factors and camouflaging. Our findings highlight key psychosocial factors as crucial targets for social change, advocating a shift from stigmatisation to acceptance and inclusion to alleviate the pressure on autistic people to camouflage.

**Lay abstract:**

Many autistic people use strategies known as ‘camouflaging’ to change how noticeable their autistic traits are in social situations. Previous research suggests that camouflaging is largely motivated by psychological and social factors. However, most studies so far have only looked at a few psychosocial factors related to camouflaging. In this study, we explored a model that included several individual psychological factors (such as fear of being negatively judged, self-esteem and autistic identity) and broader social and cultural factors (such as perceived stigma, negative life events, cultural emphasis on conformity and desire to fit in or stand out). We surveyed 225 autistic adults aged 18–77 years online. Our findings showed that several sociocultural factors were indirectly linked to camouflaging through individual psychological factors. Fear of being negatively judged emerged as a strong predictor of camouflaging. Specifically, autistic adults who perceived greater stigma, felt greater pressure to conform, had a lesser desire to stand out and a greater desire to fit in tended to experience a greater fear of being negatively judged and reported more camouflaging. In addition, those who experienced more negative life events were more likely to engage in camouflaging. Our study identifies key psychological and social factors as potential targets for social change. Our findings emphasise that our societies need to shift away from stigmatising attitudes towards accepting and including autistic people, which could reduce the pressure on autistic individuals to camouflage in social situations.

Many autistic people experience a range of psychosocial challenges, including poorer mental health (Lai, 2023), difficulties in social participation (Mason et al., 2020) and an increased likelihood of victimisation (Gibbs et al., 2021; Griffiths et al., 2019) compared with non-autistic people. To navigate these challenges, some autistic people have developed coping strategies known as camouflaging. Camouflaging involves consciously or unconsciously modifying autistic characteristics to ‘pass as normal’ to fit into non-autistic social environments (Cage & Troxell-Whitman, 2019; Hull et al., 2017). These strategies include hiding or masking one’s autistic characteristics, actively compensating for social differences by imitating non-autistic social interaction styles and attempting to assimilate with others (Hull et al., 2019). While camouflaging can be helpful and necessary for social survival in a largely non-autistic world (Bradley et al., 2021), many autistic people have described it as detrimental to their well-being and have associated camouflaging with anxiety, depression and burnout (Chapman et al., 2022; Mantzalas et al., 2022).

## Psychosocial predictors of camouflaging

Camouflaging occurs within a social context, and the existing literature highlights the role of psychosocial experiences in predicting camouflaging behaviours. Zhuang et al.’s (2023) mixed methods systematic review of 58 studies identified 3 themes relating to psychosocial correlates of camouflaging: (a) social norms and pressures of a largely non-autistic world, (b) social acceptance and rejection and (c) self-esteem and identity. In addition, Ai and colleagues (2022) have proposed a model conceptualising camouflaging as a transactional and context-dependent process motivated by psychosocial factors, such as self-esteem and stigma. Drawing from Zhuang et al. and Ai et al., we propose several psychological and sociocultural factors hypothesised to predict camouflaging, which are organised according to three levels of environmental influence (Bronfenbrenner, 1992): (a) individual psychological factors; (b) proximal sociocultural factors, encompassing direct influences from an individual’s immediate social environment and (c) distal sociocultural factors, encompassing indirect broader sociocultural influences.

## Individual psychological factors

### Fear of negative evaluation

Fear of negative evaluation (FNE) has been linked to social anxiety, with socially anxious individuals adopting safety and avoidance behaviours to conceal perceived flaws and avoid negative judgements (Moscovitch et al., 2013). Autistic people commonly experience social anxiety, with prevalence estimates ranging from 12% to 56%, significantly higher than the 7%–13% estimates for the non-autistic population (Spain et al., 2016, 2018). FNE may be implicated in camouflaging as autistic individuals often camouflage to avoid being perceived negatively, such as being labelled as ‘rude’ or ‘weird’ (Zhuang et al., 2023).

### Self-esteem

Autistic people with lower self-esteem may perceive their autistic traits as undesirable and so experience a greater need to camouflage. Zhuang et al.’s (2023) review suggests that autistic people who internalise societal stigma may develop a negative self-image over time, increasing their tendency to camouflage out of shame. Consistent with this, a quantitative study found that lower self-esteem was associated with higher self-reported camouflaging among autistic adults (Evans et al., 2023).

### Autistic identity

Autistic identity refers to a sense of affiliation with the autistic community and the integration of characteristics shared with other autistic individuals into their self-concept (Cooper et al., 2017). In 10 qualitative or mixed methods studies reviewed by Zhuang et al. (2023), participants described a trajectory in which receiving an autism diagnosis contributed to better self-understanding and acceptance of their autistic identity, resulting in less camouflaging over time. Consistent with this, one quantitative study found that autistic adults with a stronger autistic identity tended to disclose their autism diagnosis more frequently, which, in turn, was associated with reduced camouflaging (Cage, Troxell-Whitman, 2020).

## Proximal sociocultural factors

### Perceived stigma

Autistic people often experience stigmatisation across various life domains, including education (Gillespie-Lynch et al., 2019) and employment (Romualdez et al., 2021). Camouflaging can be understood as a stigma management strategy used by autistic people to conceal a stigmatised identity and protect against prejudice and discrimination (Ai et al., 2022; Botha et al., 2022; Pearson & Rose, 2021; Turnock et al., 2022). Supporting this notion, a quantitative study found that higher perceived autism stigma was associated with greater self-reported camouflaging among autistic adults (Perry et al., 2022).

### Vulnerability events

Autistic adults are more vulnerable than non-autistic adults to various negative life experiences, including victimisation, unemployment and social isolation (Gibbs et al., 2021; Griffiths et al., 2019). In 17 qualitative or mixed methods studies reviewed by Zhuang et al. (2023), autistic individuals reported being victimised due to their autistic differences, which led to increased fear of re-victimisation and potential heightened FNE, compelling them to camouflage to safeguard against further adverse experiences. Using quantitative methods, Trundle and colleagues (2022) found that autistic people who experienced greater victimisation also camouflaged more.

## Distal sociocultural factors

### Independent and interdependent self-construal

Independent and interdependent self-construal reflect the extent to which individuals perceive themselves as separate from or connected with others (Markus & Kitayama, 1991). People with a more independent self-construal tend to prioritise independence and uniqueness, while individuals with a more interdependent self-construal emphasise fitting in and connecting with others (Markus & Kitayama, 1991). Individual differences in independent and interdependent self-construals are associated with cultural orientations of individualism (prioritising personal autonomy and independence) and collectivism (emphasising group cohesion and interdependence), which are more prominent in Western and non-Western cultures, respectively (Giacomin & Jordan, 2017; Kitayama & Salvador, 2024). People with a stronger interdependent self-construal may engage in more camouflaging, driven by a greater motivation to minimise autistic differences to fit in. Conversely, those with a stronger independent self-construal may engage in less camouflaging to preserve one’s unique autistic attributes and individual autonomy.

### Cultural tightness-looseness

Different cultures vary in the strength of their social norms and the degree to which they tolerate deviation from norms (Gelfand et al., 2011). Tighter cultures tend to have stronger and clearer norms with lesser tolerance for deviation, whereas looser cultures tend to have weaker and less defined norms with greater tolerance for non-conformity (Gelfand et al., 2011). Cultural tightness-looseness is related to, but also somewhat distinct from, the concepts of individualism and collectivism, and it is possible for societies to be collectivistic and loose, or individualistic and tight (Kitayama & Salvador, 2024; Stamkou et al., 2018). Since certain autistic characteristics are often perceived as deviating from what society deems ‘normative’, autistic individuals may face stricter sanctions for their behaviours in tighter cultures. In Loo et al. (2023), one autistic interviewee described experiencing greater pressure to camouflage in Singapore compared with the United Kingdom and attributed this to Singapore’s emphasis on conformity. Thus, autistic individuals may camouflage more in culturally tighter contexts due to increased pressure to conform.

## The present study

**Figure 1.**
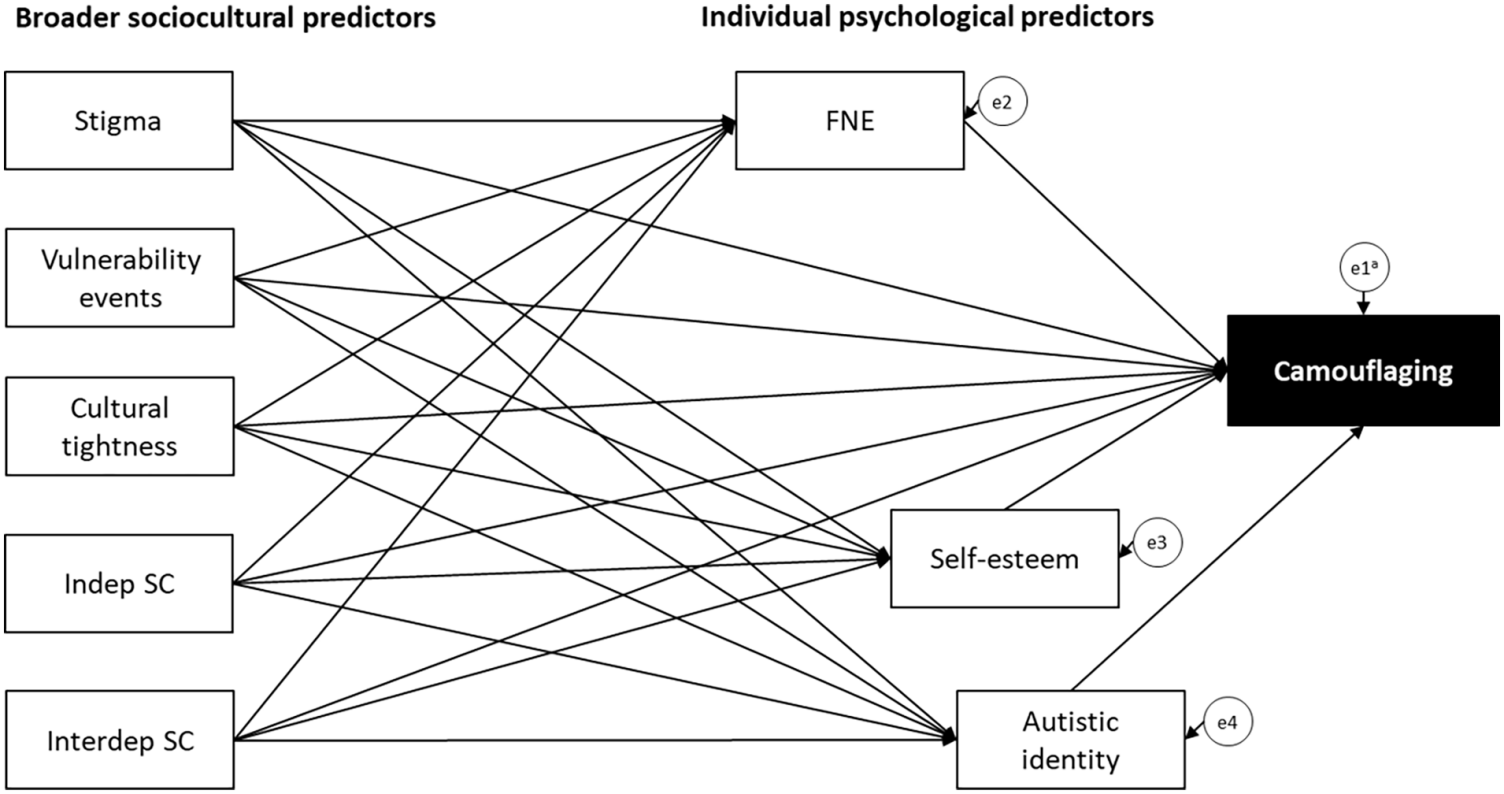
Hypothesised model. FNE: fear of negative evaluation; indep SC: independent self-construal; interdep SC: interdependent self-construal. ^a^ei are error terms.

Zhuang et al.’s (2023) systematic review found that most studies exploring psychosocial correlates of camouflaging employed qualitative methods, with only a minority (18 of 58) involving quantitative approaches. Of the few quantitative studies, most focused on biologically determined or stable individual differences (e.g. age, sex, IQ), while only a few examined a small number of psychosocial correlates (e.g. Cage, Troxell-Whitman, 2020; Perry et al., 2022). To our knowledge, no quantitative study to date has investigated an integrated theoretically and empirically informed model of psychosocial predictors of camouflaging in autistic adults. Thus, this study tested a novel model of psychosocial predictors of camouflaging derived from Zhuang et al., Ai et al. (2022) and the broader sociocultural literature discussed earlier (Figure 1).

We hypothesised that greater FNE, lower self-esteem, weaker autistic identity, greater perceived stigma, greater vulnerability to negative life events, greater cultural tightness, lower independent self-construal and higher interdependent self-construal would be associated with more camouflaging in autistic adults. In addition, as Zhuang et al.’s (2023) findings suggested that sociocultural factors may indirectly impact camouflaging through individual psychological factors, we also examined whether the hypothesised psychological factors mediated relationships between sociocultural factors and camouflaging.

## Method

### Participants

Participants were autistic adults (a) aged 18+ years, (b) able to complete an online survey in English and (c) professionally diagnosed or self-identified as autistic. Self-identified autistic individuals were included to increase the representation of those facing barriers to professional diagnoses (Lai & Baron-Cohen, 2015; Lewis, 2017) or not formally identified due to camouflaging (Libsack et al., 2021). Moreover, research has shown no statistically significant differences between professionally diagnosed and self-identified autistic participants in self-reported autistic traits, autism identity, self-esteem and stigma experiences (English et al., 2021; McDonald, 2020).

Recruitment occurred between May and August 2021 through advertisements distributed via social media, autism associations and Prolific (https://prolific.co; a U.K.-based online research participation platform). Of the 259 respondents who accessed our survey, we excluded 32 who failed our screening checks (see Procedure). In addition, data from two respondents were identified as influential multivariate outliers and excluded from subsequent analyses. Our final sample comprised 225 autistic adults aged 18–77 years (*M* = 35.5, *SD* = 11.8).

Most participants were female, White, employed, university-educated and residing in Australia (Table 1). About 70% of participants reported having a mental health diagnosis in the last 5 years, most commonly anxiety and depression. Most were professionally diagnosed (*n* = 183; 81.3%), while 42 (18.7%) self-identified as autistic. In our study, there was no significant difference in Broad Autism Phenotype Questionnaire (BAPQ) total scores (Hurley et al., 2007) between participants with professional diagnoses (*M* = 4.3, *SD* = 0.6) and those self-identifying as autistic (*M* = 4.4, *SD* = 0.5; *t*(223) = 0.61, *p* = 0.546, *d* = 0.10). Most (*n* = 217; 96.4%)^1^ scored above the BAPQ total cut-off score of 3.15, previously established as indicating a high likelihood of clinically significant levels of autistic traits (Hurley et al., 2007). Among professionally diagnosed participants, the mean age at autism diagnosis was 27.5 years (*SD* = 14.2, range 2–61).

**Table 1.** Participant characteristics (*N* = 225).

Characteristics	*N*	%
Gender		
Female	137	60.9
Male	64	28.4
Other genders^a^	21	9.3
Preferred not to say	3	1.3
Ethnicity		
White	193	85.8
Asian	13	5.8
Multiethnic	13	5.8
Black/African/Caribbean	4	1.8
Hispanic/Latino	2	0.9
Country of residence		
Australia	125	55.6
United Kingdom	45	20.0
United States of America	30	13.3
New Zealand	8	3.6
Canada	7	3.1
Other European countries	7	3.1
Asian countries (Singapore, Hong Kong)	3	1.3
Highest educational level		
Undergraduate degree	76	33.8
Postgraduate degree	58	25.8
Secondary school	50	22.2
Trade/technical certificate	34	15.1
Primary school	3	1.3
Other qualifications (e.g., diploma, A-level certificate)	4	1.8
Employment status		
Employed full-time	63	28.0
Employed part-time	50	22.2
Unemployed	42	18.7
Student	36	16.0
Homemaker/caregiver	19	8.4
Unpaid work (e.g., volunteering, unpaid internship)	8	3.6
Retired	5	2.2
Preferred not to say	2	0.9
Living arrangements		
Living with partner and/or child(ren)	103	45.8
*Living* with parents/relative(s)	59	26.2
Living alone	40	17.8
Living with friend(s)/housemate(s)	21	9.3
Other	2	0.9
Other neurodevelopmental diagnoses		
Attention-deficit/hyperactivity disorder	55	24.4
Specific learning disorder	12	5.3
Intellectual disability	3	1.3
Other (e.g., cerebral palsy, developmental coordination disorder)	3	1.3
Preferred not to say	6	2.7
Mental health diagnoses (in the last five years)		
Anxiety	127	56.4
Depression	113	50.2
Post-traumatic stress disorder	29	12.9
Obsessive-compulsive disorder	12	5.3
Personality disorders	7	3.1
Bipolar disorder	5	2.2
Eating disorders	5	2.2
Other (e.g., psychotic disorders, dissociative disorders)	10	4.4
Preferred not to say	6	2.7

a Other genders included non-binary (*n* = 12), agender (*n* = 3), genderqueer (*n* = 2), gender neutral (*n* = 1), gender fluid (*n* = 1), gender curious (*n* = 1), and demifemale (*n* = 1).

### Procedure

Ethical approval was granted by the University of Western Australia Human Research Ethics Committee (reference number: 2021/ET000065).

This study is part of a larger project exploring psychosocial factors associated with camouflaging, mental health and burnout in autistic adults across three time points. Participants completed demographic questions and self-report measures on a Qualtrics-hosted survey (https://www.qualtrics.com), taking approximately 45–60 min. All participants provided written informed consent and could choose ‘prefer not to say’ for all questions. Participants received AUD20 reimbursement for their time.

Given that this was an online survey, we employed several strategies to reduce invalid responses and enhance data quality. Initially, participants were invited to express their interest by completing a brief questionnaire. Eligible participants who achieved a reCAPTCHA score of ⩾ 0.70^2^ received an individual survey link via email. Our study survey included two CAPTCHA questions and two attention check items. Upon completion, participants were asked to indicate whether their data was valid for research. We excluded the data of 32 participants for the following reasons: (a) incomplete or unsubmitted (*n* = 17), (b) ineligibility (*n* = 6), (c) a reCAPTCHA score of < .5 (*n* = 3), (d) indicated invalid data (*n* = 1), (e) chose ‘prefer not to say’ for over 20% of responses across all measures (*n* = 2) and (f) chose ‘prefer not to say’ for all items for at least one measure (*n* = 3).

### Measures

#### Autistic traits

The 36-item BAPQ (Hurley et al., 2007) measures autism-related characteristics (rated from 1 = *very rarely* to 6 = *very often*; mean-item total score range 1–6) and demonstrates good sensitivity, specificity and excellent internal consistency in autistic and non-autistic groups (α = 0.95–0.96; Hull et al., 2019; Hurley et al., 2007; Sasson et al., 2013).

#### Camouflaging

The 25-item Camouflaging Autistic Traits Questionnaire (CAT-Q; Hull et al., 2019) measures camouflaging behaviours (rated from 1 = *strongly disagree* to 7 = *strongly agree*; total score range 25–175). It has been validated in autistic and non-autistic adults, demonstrating equivalence in factor structure across gender and diagnostic groups and high internal consistency (α = 0.91–0.94; Hannon et al., 2023; Hull et al., 2019).

#### Individual psychological factors

##### FNE

The eight-item Brief Fear of Negative Evaluation-II Scale (BFNE-II; Carleton et al., 2007) measures apprehension related to negative evaluation by others (rated from 0 = *not at all characteristic of me* to 4 = *extremely characteristic of me*; total score range 0–32). It demonstrates moderate convergent and discriminant validity and excellent internal consistency in clinical and nonclinical populations (α = 0.95–0.96; Carleton et al., 2007, 2011).

##### Self-esteem

The 10-item Rosenberg Self-Esteem Scale (RSES; Rosenberg, 1965) measures global self-worth (rated from 1 = *strongly disagree* to 4 = *strongly agree*; total score 10–40). It demonstrates excellent internal consistency among autistic adults (α = 0.90–0.92; Cooper et al., 2017; Corden et al., 2021; Nguyen et al., 2020).

##### Autistic identity

The 14-item Multicomponent In-group Identification Scale (MIIS; Leach et al., 2008) measures in-group identification (rated from 1 = *strongly disagree* to 7 = *strongly agree*; mean-item total score range 1–7). It has been adapted to measure autism identification with good to excellent internal consistency (α = 0.84–0.91; Cooper et al., 2017, 2020, 2022; Maitland et al., 2021). Our adaptation used the terms ‘autistic individual/person/community’.

#### Proximal sociocultural factors

##### Perceived stigma

The original five-item Perceived Stigma Measure (PSM; Shtayermman, 2009) assesses perceived stigma related to having Asperger’s syndrome (a former autism diagnostic term). In this study, we replaced ‘having Asperger’s syndrome’ with ‘being autistic/on the autism spectrum’, to align with updated terminology and many autistic adults’ preferences (Bury et al., 2020; Kenny et al., 2016). To capture a broader range of responses, we modified the rating scale from the original ‘yes/no’ to a 5-point Likert-type scale (0 = *never*; 4 = *very often*; total score range 0–20). To our knowledge, the PSM is the only available measure directly assessing perceived autism stigma in the literature. Our adaptation demonstrated good internal consistency in our dataset (Table 2).

**Table 2.** Descriptive statistics, reliabilities and correlations among study variables (*N* = 225).

	Camouflaging	FNE	Self-esteem	Autistic identity	Stigma	Vulnerability events	Cultural tightness	Indep SC	Interdep SC
FNE	0.40***								
Self–esteem	–0.09	–0.47***							
Autistic identity	0.21**	0.12	0.28***						
Stigma	0.11	0.21**	–0.21**	–0.02					
Vulnerability events	0.26***	0.12	–0.14*	0.03	0.32*** [Table-fn table-fn3-13623613241262110]				
Cultural tightness	0.16*	0.16*	0.11	0.15*	0.05	0.05			
Indep SC	–0.20**	–0.40***	0.58***	0.25***	–0.12	–0.01	0.09		
Interdep SC	0.13	0.23***	–0.10	0.15*	–0.03	–0.08	0.19**	–0.09	–
*M*	132.72	22.48	24.14	4.73	13.37	22.32	4.79	4.32	4.43
*SD*	20.62	8.68	6.06	1.14	4.49	10.12	0.74	0.82	0.83
Range	73–175	0–32	10–40	1.50–7.00	0–20	3–48	2.60–6.00	1.92–6.67	2.35–6.83
Cronbach’s alpha (α)	0.89	0.95	0.89	0.91	0.84	0.91	0.65	0.71	0.72

FNE: fear of negative evaluation; indep SC: independent self-construal; interdep SC: interdependent self-construal; *SD*: standard deviation.

* *p* < 0.05; **p < 0.01; ***p < 0.001; correlations ⩾ 0.3 in bold.

##### Vulnerability events

The 60-item Vulnerability Experiences Quotient (VEQ; Griffiths et al., 2019) assesses the frequency of negative experiences across multiple life domains. Participants scored 1 for presence and 0 for absence of each experience, except for three social support items, which are reverse scored (total score range 0–60). The VEQ demonstrates very good internal consistency among autistic adults (α = 0.89; Griffiths et al., 2019).

#### Distal sociocultural factors

##### Cultural tightness-looseness

The six-item Tightness-Looseness Scale (TLS; Gelfand et al., 2011) assesses the strength of social norms and tolerance for norm violations (rated from 1 = *strongly disagree* to 6 = *strongly agree*; mean-item total score range 1–6). It demonstrates good validity and internal consistency in large cross-national studies (α = 0.80–0.85; Aktas et al., 2016; Eriksson et al., 2021; Gelfand et al., 2011).

##### Independent and interdependent self-construal

The Self-Construal Scale (SCS; Singelis, 1994) comprises a 12-item independent subscale and a 12-item interdependent subscale (rated from 1 = *strongly disagree* to 7 = *strongly agree*; mean-item total subscale score range 1–7). Both subscales have acceptable internal consistency in ethnically diverse student populations (α = 0.69–0.81; Christopher & Skillman, 2009; Singelis, 1994; Singelis et al., 1995).

### Data analyses

IBM SPSS Statistics version 29.0 and AMOS 29.0 were used.

#### Missing data

Responses indicating ‘prefer not to say’ were treated as missing data, which ranged from 0.1% to 3.3% across measures, with an overall 0.8% missing values. Little’s MCAR test indicated that data were missing completely at random for all measures, except for the CAT-Q, BFNE and TLS. Item-level missing data were addressed using multiple imputation (Woods et al., 2023). Twenty imputed datasets were combined into a single pooled dataset using the ‘bar procedure’ (Baranzini, 2018) for subsequent analyses.

#### Main analyses

To examine direct and indirect relationships between variables, path analysis was conducted in AMOS using maximum likelihood estimation and 10,000 bootstrap samples. Mediation was considered significant if the bootstrapped 95% bias-corrected confidence interval for the indirect effect excluded 0 (Hayes, 2009).

The hypothesised model (Figure 1) was assessed for goodness of fit. Modification indices were examined to consider the inclusion of additional, theoretically viable relationships. Model fit was assessed using multiple indices, with the following cut-off values indicating a good fit: comparative fit index (CFI) ⩾ 0.95, standardised root mean square residual (SRMR) ⩽ 0.08 and root mean square error of approximation (RMSEA) ⩽ 0.06 (Hu & Bentler, 1999). Model trimming was performed by removing non-significant paths to obtain the most parsimonious model.

### Authors’ positionality

Our approach was influenced by our training in psychology, professional experiences as academics and lived experiences as autistic individuals. Our research team holds professional and personal values that align with a neurodiversity-affirming lens and the social model of disability.

### Community involvement

The larger research project was developed in consultation with an autistic advisor who provided input on the conceptualisation, relevance of psychosocial factors and appropriateness of the survey. For this study, we engaged two additional autistic advisors who contributed to the model development, measure selection and interpretation of findings, ensuring alignment with their lived experiences and understanding of camouflaging within the autistic community. All advisors were reimbursed for their time and are co-authors.

## Results

Descriptive statistics, internal consistencies and intercorrelations are presented in Table 2. FNE, vulnerability events, autistic identity and cultural tightness were significantly positively correlated with camouflaging, while independent self-construal was significantly negatively correlated with camouflaging, with small to medium correlations.^3^ Internal consistencies ranged from acceptable to excellent, except for TLS which had borderline reliability.^4^ Mardia’s (1970, 1974) normalised estimate of multivariate kurtosis was 1.43, lower than the recommended cut-off value of 5.0 (Bentler, 2006; Byrne, 2016), indicating multivariate normality.

The initial model demonstrated inadequate fit, χ^2^(13) = 90.85, *p* < 0.001, CFI = 0.76, SRMR = 0.09, RMSEA = 0.16 (90% CI (0.13, 0.20)). Guided by modification indices and previous literature on relationships between the variables, covariances were specified between stigma and vulnerability events,^5^ cultural tightness and interdependent self-construal^6^ as well as among the error terms of FNE, self-esteem and autistic identity.^7^

**Figure 2.**
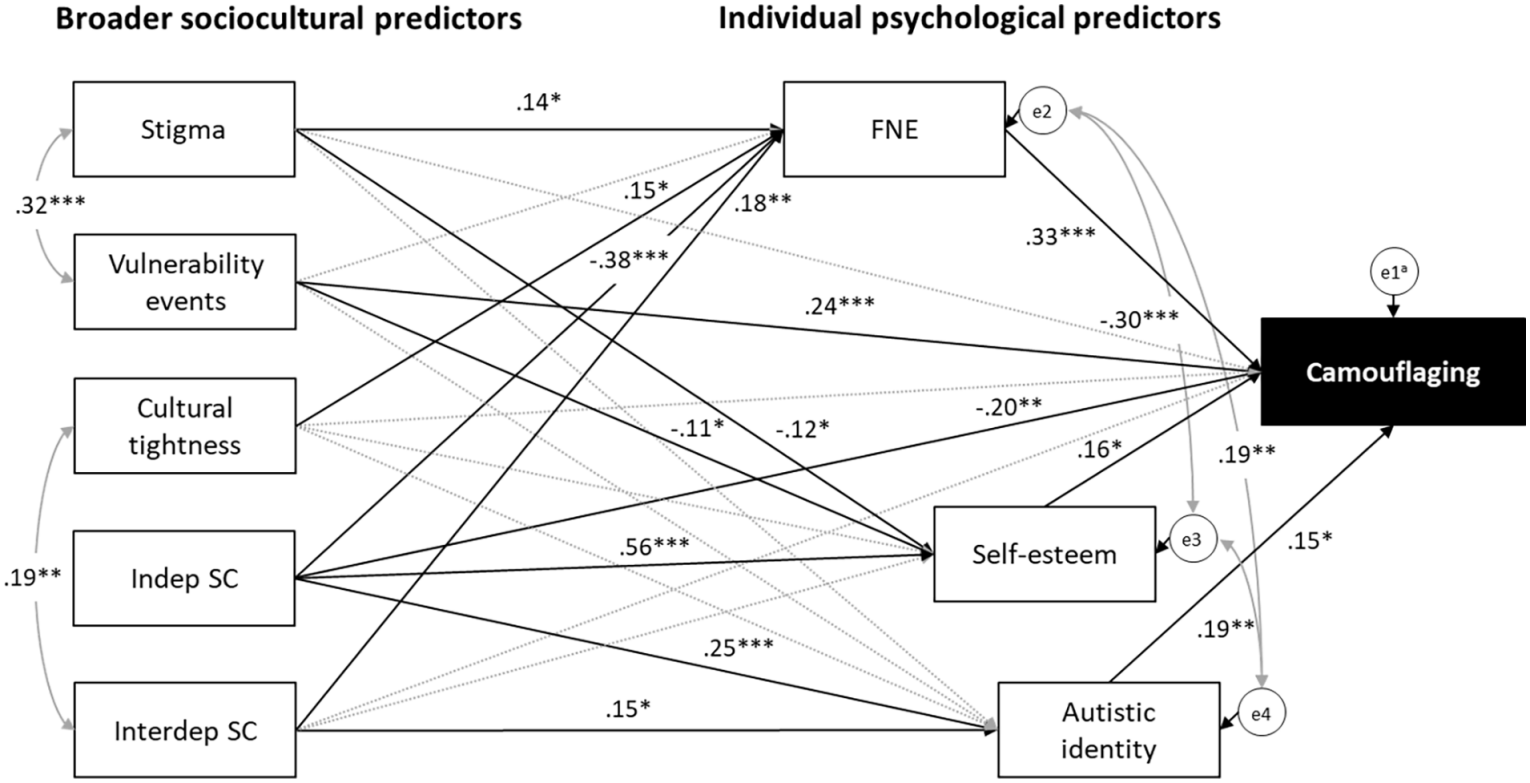
Revised model with post-hoc modifications. Bold lines indicate significant paths; dotted lines indicate non-significant paths. Grey lines indicate paths added from post hoc modifications. Standardised coefficients are indicated for significant paths only. FNE: fear of negative evaluation; indep SC: independent self-construal; interdep SC: interdependent self-construal. ^a^ei are error terms. *p < 0.05; **p < 0.01; ***p < 0.001.

The revised model (Figure 2) exhibited excellent fit, χ^2^(8) = 11.29, *p* = 0.186, CFI = 0.99, SRMR = 0.04, RMSEA = 0.04 (90% CI (0.00, 0.10)), accounting for 26.5% of the variance in camouflaging. To derive the most parsimonious model, model trimming of the revised model was conducted by iteratively removing non-significant paths one by one, starting with the path with the largest *p*-value. The model was rerun to examine changes in model fit indices and to identify the next least significant path for removal until all remaining paths in the model were significant. In total, 10 paths were removed, including 3 direct paths from hypothesised predictors to camouflaging and 7 mediating paths between the predictors.

**Figure 3.**
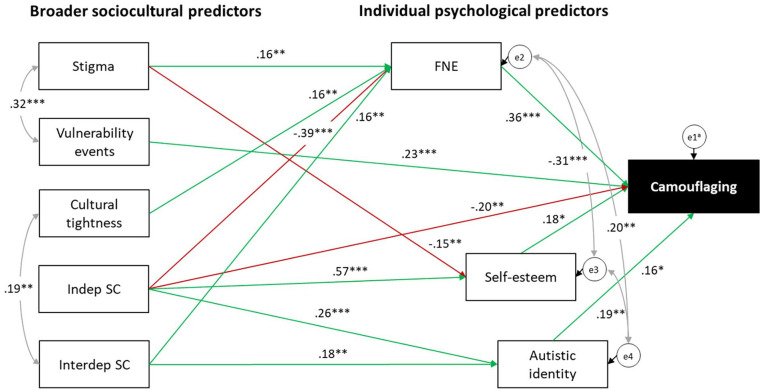
Final trimmed model. Green lines indicate positive relationships; red lines indicate negative relationships. Standardised coefficients are reported. FNE: fear of negative evaluation; indep SC: independent self-construal; interdep SC: interdependent self-construal. ^a^ei are error terms. *p < 0.05; **p < 0.01; ***p < 0.001.

The final trimmed model (Figure 3) maintained excellent fit, χ^2^(18) = 22.98, *p* = 0.191, CFI = 0.98, SRMR = 0.05, RMSEA = 0.04 (90% CI (0.00, 0.07)), explaining 24.9% of the variance in camouflaging. Greater vulnerability events and lower levels of independent self-construal were directly associated with increased camouflaging. Perceived stigma, cultural tightness and both independent and interdependent self-construal were indirectly associated with camouflaging through individual psychological factors. As expected, greater FNE was associated with more camouflaging, but unexpectedly, higher self-esteem and a stronger autistic identity were linked to greater camouflaging. All statistically significant mediating paths before and after model trimming are presented in Table 3.

**Table 3.** Statistically significant mediating paths (*N* = 225).

Mediating paths	Before model trimming					After model trimming				
	*b* (*SE*)	95% CI		*p*	β^a^	*b (SE)*	95% CI		*p*	β^a^
		*LL*	*UL*				*LL*	*UL*		
1. Stigma → FNE → Cam	0.22 (0.12)	0.03	0.51	0.024	0.05	0.26 (0.12)	0.07	0.54	0.006	0.06
2. Stigma → Self-esteem → Cam	–0.09 (0.07)	–0.29	–0.001	0.045	–0.02	–0.12 (0.08)	–0.33	–0.02	0.016	–0.03
3. Cultural tightness → FNE → Cam	1.40 (0.63)	0.42	2.99	0.006	0.05	1.60 (0.64)	0.57	3.19	0.002	0.06
4. Indep SC → FNE → Cam	–3.20 (0.93)	–5.21	–1.56	<0.001	–0.13	–3.41 (0.90)	–5.39	–1.83	<0.001	–0.14
5. Indep SC → Self–esteem → Cam	2.23 (1.06)	0.23	4.41	0.031	0.09	2.48 (1.09)	0.44	4.78	0.019	0.10
6. Indep SC → Autistic identity → Cam	0.93 (0.47)	0.20	2.08	0.011	0.04	1.02 (0.48)	0.27	2.20	0.007	0.04
7. Interdep SC → FNE → Cam	1.51 (0.64)	0.51	3.04	0.001	0.06	1.39 (0.58)	0.46	2.77	0.002	0.06
8. Interdep SC → Autistic identity → Cam	0.56 (0.38)	0.05	1.58	0.021	0.02	0.70 (0.41)	0.11	1.80	0.011	0.03

*SE*: standard error; CI: confidence interval; LL: lower limit; UL: upper limit; FNE: fear of negative evaluation; cam: camouflaging; indep SC: independent self–construal; interdep SC: interdependent self-construal.

a As AMOS provides only unstandardised specific indirect effects, standardised specific indirect effects were manually calculated by multiplying the standardised path coefficients associated with each indirect effect (MacKinnon et al., 2007).

## Discussion

To our knowledge, this is the first study to examine an integrated model of psychosocial predictors of camouflaging in autistic adults. Results partially supported the hypothesised model, which demonstrated excellent model fit after post hoc modifications. The final model accounted for about 25% of the variance in camouflaging, demonstrating a substantial contribution from psychosocial factors.

Several sociocultural factors – perceived stigma, cultural tightness and interdependent self-construal – were not directly associated with camouflaging; instead, they were indirectly associated with camouflaging through individual psychological factors, such as FNE, self-esteem and autistic identity. These findings align with the transactional model of camouflaging proposed by Ai and colleagues (2022) and empirically support conclusions from Zhuang et al.’s (2023) review, suggesting that sociocultural factors may indirectly influence camouflaging through autistic individuals’ internalisation of societal stigma and norms.

Notably, vulnerability to negative life events was the only sociocultural factor that was directly associated with camouflaging, without mediation by individual psychological factors. This could be attributed to camouflaging potentially being a conditioned trauma response driven by autistic people’s need for safety and social survival and directly impacted by negative life experiences, including victimisation, discrimination and abuse (Chapman et al., 2022; Pearson et al., 2022; Seers & Hogg, 2022). Given the cross-sectional nature of our data, it is also possible that the relationship between camouflaging and vulnerability to negative events may be bidirectional. While many autistic people camouflage to reduce or avoid victimisation and other negative life experiences, camouflaging may potentially increase one’s risk of being victimised through heightened compliance and a desire to please others (Pearson et al., 2022; Zhuang et al., 2023).

FNE emerged as a strong and important predictor of camouflaging, mediating most relationships between broader sociocultural factors and camouflaging. Autistic adults perceiving greater autism stigma and cultural tightness reported increased FNE, which, in turn, was associated with more camouflaging. These results extend Perry et al.’s (2022) findings by highlighting the mediating role of FNE in the relationship between autism stigma and camouflaging. They are also consistent with accounts of autistic individuals who camouflage to mitigate prejudicial biases (Zhuang et al., 2023). Moreover, our findings align with the literature indicating that tighter cultures are associated with a heightened sense of evaluation by others, promoting self-monitoring and behavioural mimicry (Baldwin & Mussweiler, 2018).

Given that FNE is a well-established mechanism of social anxiety (Rapee & Heimberg, 1997; Zhang et al., 2023), the statistically strong association between FNE and camouflaging may, to some extent, reflect a high degree of co-occurring social anxiety among our participants. Indeed, social anxiety is a common co-occurring mental health condition among autistic individuals (Spain et al., 2016, 2018). Furthermore, recent research has shown a strong positive association between camouflaging and social anxiety while also demonstrating construct overlap between camouflaging and social anxiety-related safety behaviours (Lei et al., 2024). Autistic people with greater FNE may, therefore, camouflage more to safeguard against potential adverse experiences and to cope with their social anxiety (Lei et al., 2024; Spain et al., 2020).

Consistent with our hypothesis, autistic adults with a stronger interdependent self-construal reported greater FNE and camouflaged more. In contrast, those with a stronger independent self-construal reported lower FNE and camouflaged less. Individuals with a stronger interdependent self-construal tend to value social harmony, likely increasing their FNE and motivation to camouflage to conform to social norms (Okawa et al., 2021; Tveleneva et al., 2023). Conversely, those with a stronger independent self-construal likely prioritise uniqueness and autonomy, likely reducing their FNE and motivation to conform (Okazaki, 1997; Torelli, 2006). Zhuang et al. (2023) discussed two potential trajectories involving continued camouflaging versus a shift from camouflaging towards disclosure and self-advocacy, which could be explained by self-construal differences. Autistic individuals with a stronger independent self-construal may be less concerned about social evaluations and have a greater desire to assert their individuality, thus being more inclined to utilise disclosure and self-advocacy instead of camouflaging. Conversely, those with a stronger interdependent self-construal may be more concerned with others’ judgements and motivated to camouflage to fit in. While disclosure and self-advocacy were not measured in this study, they are likely compatible with camouflaging and are not mutually exclusive pathways. Autistic individuals may select camouflaging, diagnostic disclosure and self-advocacy as goal-directed behaviours used in different contexts and settings to assert their agency in a non-autistic world (Zhuang et al., 2023).

Contrary to our hypothesis, the final mediation model showed a positive association between self-esteem and camouflaging. Interestingly, the zero-order correlation between self-esteem and camouflaging was negative (Table 2), albeit non-significant, so the change in direction and significance in the model suggests the presence of a suppressor effect. Ai et al. (2022) proposed two routes to camouflaging: one seeking positive experiences (‘voluntary self-enhancement’) and another avoiding negative experiences (‘compelled social modification’, p. 3). The variance in camouflaging explained by negatively-valenced psychosocial predictors (e.g. FNE, stigma, vulnerability events) may be related to ‘compelled social modification’, where marginalised autistic people feel compelled to camouflage for safety. After accounting for this variance, the remaining variance in camouflaging may be related to ‘voluntary self-enhancement’, showing a positive association with self-esteem. As part of ‘voluntary self-enhancement’, autistic people with higher self-esteem may assert agency by camouflaging for pragmatic purposes, such as accessing opportunities (Ai et al., 2022; Zhuang et al., 2023). This dynamic could create a reinforcing loop where the positive outcomes of camouflaging contribute to higher self-esteem, motivating continued camouflaging. Conversely, autistic adults in our study who perceived greater autism stigma reported lower self-esteem and, in turn, reduced camouflaging. Greater awareness of public stigma may lead autistic individuals to internalise stigmatising attitudes, resulting in self-stigma, lowered self-esteem and the ‘why try’ effect (Corrigan et al., 2009, 2016) – a sense of futility and diminished motivation to pursue goals. Given that camouflaging can be a goal-directed behaviour that is effortful and exhausting (Zhuang et al., 2023), autistic individuals experiencing the ‘why try’ effect may be less motivated to engage in ‘voluntary self-enhancement’ and camouflage less. These findings highlight the complex and dynamic interplay between camouflaging and psychosocial factors, providing insight into the intricacies of camouflaging decisions as autistic individuals weigh up the benefits and costs of camouflaging versus not camouflaging across different contexts.

Finally, another unexpected finding was that a stronger autistic identity was associated with increased camouflaging, both as a zero-order association and in the final mediation model. This finding contradicts Zhuang et al.’s (2023) conclusions, which suggested that a stronger autistic identity may contribute to reduced camouflaging. However, they align with Cage and Troxell-Whitman’s (2020) study demonstrating a positive relationship between camouflaging and a stronger autistic identity. Individuals with a stronger autistic identity may have a greater awareness of their autistic traits and related stigma, potentially motivating them to camouflage (Cage et al., 2022).

### Strengths, limitations and future directions

A notable strength of our study lies in the examination of an integrated, comprehensive model encompassing various psychological and sociocultural predictors of camouflaging. This approach revealed unexpected associations between some of the psychosocial factors examined and camouflaging, such as the positive correlation between camouflaging and self-esteem in the final model, which would not have been revealed in simple zero-order correlations. This study’s findings support our understanding of camouflaging as a complex phenomenon with context-dependent motivations and pathways. We encourage future research to similarly adopt an integrated model approach to understanding camouflaging, so that a more comprehensive, in-depth understanding of complex influences and interactions can be achieved.

Our study also has several limitations. Considering the exploratory nature of our model and the use of trimming procedures in a single sample, our findings should be considered tentative. Our participants were self-selected and recruited online, introducing sampling bias and limiting the generalisability of our findings to the wider autistic population (Rødgaard et al., 2022). Most participants were female, White, employed, university-educated, without intellectual disabilities and received late autism diagnoses; thus, future studies should test our model in independent cohorts with more diverse characteristics.

Furthermore, several psychosocial measures used have not been specifically validated or developed for the autistic population. Moreover, our study was cross-sectional, so we could not test directional paths among variables. While factors such as FNE, vulnerability events and self-esteem may influence camouflaging, it is plausible that camouflaging also reinforces these psychosocial experiences. Prospective longitudinal studies are needed to clarify the nature and direction of the relationships between psychosocial factors and camouflaging.

While our study demonstrated that the hypothesised psychosocial factors predicted a substantial amount of variance in camouflaging (25%), a large portion of variance remains unexplained. Future studies could explore additional predictors of camouflaging, including psychosocial factors not investigated in our study but considered relevant by Zhuang et al. (2023) and Ai et al. (2022), such as social motivation and internalised stigma. Finally, future research should explore whether specific psychosocial motivations for camouflaging are associated with more positive or negative mental health outcomes, which may provide insights into mitigating any adverse effects on mental well-being. Specifically, studies can explore the link between camouflaging, FNE and social anxiety, given that autistic people, driven by FNE, may engage in camouflaging as a safety behaviour to cope with social anxiety, potentially perpetuating social anxiety in the long run (Lei et al., 2024; Spain et al., 2020).

### Implications

Our findings underscore the impact of social and contextual factors, including social stigma and norms, on how autistic individuals perceive themselves and relate to others, influencing their engagement in camouflaging. Drawing on Bronfenbrenner’s (1992) ecological systems theory, a nuanced understanding of camouflaging acknowledges that it is shaped by the interplay between autistic individuals and their social environment, rather than stemming solely from their intrinsic traits (Ai et al., 2022). Applying the minority stress model, autistic people can be seen as an identity-based minority facing stigma-related stressors, including everyday discrimination, internalised stigma and camouflaging, which negatively affect mental well-being (Botha & Frost, 2020). Given our finding that camouflaging is strongly influenced by FNE, interventions aimed at shifting societal attitudes away from stigmatisation and towards acceptance and inclusion of autistic people may be important in reducing psychosocial pressures to camouflage.

While social change is crucial to alleviate pressure on autistic people to camouflage, cultural and societal shifts in attitudes take time. Consequently, until such shifts occur, camouflaging serves as a sophisticated set of strategies employed by many autistic individuals to navigate and negotiate their social realities. Despite its consistent links with poorer mental health (e.g. Beck et al., 2020; Hull et al., 2019, 2021; Zhuang et al., 2023), autistic individuals who do not camouflage or are ‘unsuccessful’ in doing so may risk exposure to adverse psychosocial responses, negatively impacting well-being (Lai, 2023). Beyond serving as a defensive response to stigma and social pressures, our findings suggest that camouflaging may also be a goal-directed behaviour used by some autistic individuals with higher self-esteem to assert their agency in a non-autistic world. Considering the complexities surrounding camouflaging motivations, professionals working with autistic people should aim to understand and explore individual reasons, contexts and functions of camouflaging and prioritise the agency and autonomy of autistic individuals in decisions about when, where, with whom and how to camouflage. Rather than encouraging autistic people to simply ‘drop their mask’ and stop camouflaging, professionals should support them in weighing the benefits and costs of camouflaging against alternative strategies, such as disclosure and self-advocacy across various contexts. Relatedly, psychosocial interventions should address external social factors contributing to camouflaging, such as bullying or stigmatising narratives, while supporting autistic individuals in choosing environments that suit their needs and provide a better person-environment fit.

## Notes

1. Of the eight participants scoring below the BAPQ cut-off score, seven were professionally diagnosed, and one self-identified as autistic. Path analyses were conducted twice: once including and once excluding participants scoring below the BAPQ cut-off score, and the pattern of results was largely similar. Since most of these participants were professionally diagnosed, and findings showed minimal difference when they were excluded, results are presented including them in the analyses (*N* = 225).

2. According to Qualtrics (n.d.), a reCAPTCHA score of 0.5 and above signals a likely human respondent, while a score below 0.5 suggests a likely bot. During screening, we adopted a stricter criterion of a reCAPTCHA score of 0.7 and above to minimise the risk of inviting bots to participate in our study.

3. Following Cohen’s (1988, 1992) guidelines, Pearson’s *r* values of 0.1, 0.3 and 0.5 represent small, medium and large correlations, respectively.

4. Item 4 of the TLS ( ‘People in this country have a great deal of freedom in deciding how they want to behave in most situations’) was excluded due to its low item-total correlation of 0.1. Its removal improved Cronbach’s alpha from 0.58 to 0.65. Being the only reverse-coded item, this item may have introduced construct-unrelated method variance and was shown to have the lowest item loading in the original article by Gelfand et al. (2011).

5. Stigma-related stressors increase autistic people’s vulnerability to negative life experiences, such as victimisation, unemployment, poverty, isolation and lack of social support (Botha & Frost, 2020; Frost, 2011).

6. Interdependent self-construal tends to be more prevalent in tighter cultures (Carpenter, 2000; Triandis, 1989).

7. Self-esteem has been found to be positively associated with autistic identity (Cooper et al., 2017; Corden et al., 2021) and negatively associated with FNE (Cheng et al., 2015; Gök & Yalçınkaya-Alkar, 2023). To our knowledge, no study has specifically examined the relationship between FNE and autistic identity. However, given that FNE has been found to be positively associated with autistic traits (Spain et al., 2016), a relationship between FNE and autistic identity is plausible.
